# Differentiating Myelography Contrast from Intraventricular and Subarachnoid Hemorrhage Using Dual-Energy CT of the Head: A Case Report and a Review of Literature

**DOI:** 10.7759/cureus.67416

**Published:** 2024-08-21

**Authors:** James Garda, Sarah-Marie C Gonzalez, Harold Sonnier, Awais Z Vance

**Affiliations:** 1 Neurological Surgery, Baylor College of Medicine, Temple, USA; 2 Neurological Surgery, Baylor Scott and White Medical Center - Temple, Temple, USA; 3 Radiology, Baylor Scott and White Medical Center - Temple, Temple, USA

**Keywords:** subarachnoid hemorrhage, iodine contrast, intraventricular hemorrhage, ct scan head, dual energy computed tomography

## Abstract

Single-energy computed tomography (SECT) head is a common diagnostic tool to evaluate for intracranial hemorrhage in emergency settings due to its widespread accessibility and non-invasive nature. However, SECT has densitometric evaluation limitations. For example, hyperdensities on SECT such as blood product and iodine contrast appear similarly. Dual-energy CT (DECT) is a relatively under-utilized imaging modality that has the capability to differentiate between multiple materials. This imaging technique can be extremely useful in identifying materials that are otherwise indistinguishable from standard SECT.

The authors present a case of a patient with findings suspicious of intraventricular and subarachnoid hemorrhage on conventional SECT. The suspected hemorrhage was subsequently ruled out utilizing DECT, as iodinated contrast can be subtracted out, yielding an image that can differentiate iodine contrast from blood or other hyperdense material. The authors discuss the underlying physics, potential advantages, and limitations of the DECT.

## Introduction

Contrast extravasation into the subarachnoid space is a well-known occurrence following the administration of intra-arterial contrast during the endovascular intervention of large vessel occlusions. Contrast extravasation in this setting is thought to result from blood-brain barrier breakdown by reperfusion injury [1-5]. There have been few reports of contrast extravasation into the subarachnoid space following intravenous administration, but of those documented, there were alternative explanations for blood-brain barrier breakdown, i.e. intracranial tumor or trauma [6]. The hyperattenuation of contrast staining can appear similarly to blood product on a conventional single-energy computed tomography (SECT) and may lead to an inaccurate diagnosis and subsequent inappropriate and potentially harmful treatment. Dual-energy computed tomography (DECT) has been a useful tool to distinguish iodine contrast staining from hemorrhage in various clinical applications [6].

DECT is an imaging technique that utilizes two different X-ray energy spectra [7]. Changes in the attenuation of materials at different energy levels allow DECT to characterize and differentiate materials, reduce artifacts, and often improve contrast-to-noise and signal-to-noise ratios over SECT. Using this technology, iodinated contrast can be subtracted out, yielding a DECT subtraction image that can differentiate iodine contrast from blood or other hyperdense material [8].

The aim of this case report is to illustrate the diagnostic utility of DECT to quickly and reliably differentiate contrast extravasation from subarachnoid hemorrhage (SAH) and intraventricular hemorrhage (IVH) in a patient who had no known major risk factors, signs, or symptoms to suggest either hemorrhage or contrast extravasation.

## Case presentation

The patient was a 74-year-old Caucasian male with a history of hypertension, hyperlipidemia, poorly controlled type 2 diabetes mellitus (DM), alcohol abuse, and post-traumatic stress disorder (PTSD). He was initially admitted to a Veterans Affairs (VA) hospital for diabetic ketoacidosis and pneumonia before transferring to a nearby private hospital for treatment of an unspecified compressive myelopathy. There was no access to medical records from this hospital, and the patient was unable to recall specific treatment he had received at the private hospital. After discharge, he presented back to the VA hospital for confusion and cognitive decline, where a CT head was acquired, reportedly showing hyperdensities consistent with IVH and SAH, as well as diffuse cerebral edema. The radiographic findings in the context of his acute confusion and progressive cognitive decline led to his transfer to our institution for a higher level of care.

Upon his presentation to the tertiary care center, the patient’s confusion had resolved. The patient was awake with normal affect, alert, and oriented to self, place, time, and situation, Glasgow Coma Scale (GCS) 15, and without any focal neurologic deficits. The patient had a left-sided scalp hematoma of unknown origin with no active external bleeding. He denied recent falls, headaches, nausea, vomiting, changes in vision, new numbness, weakness, or bowel and bladder issues. Vital signs were normal besides an elevated blood pressure of 179/85.

**Figure 1:**
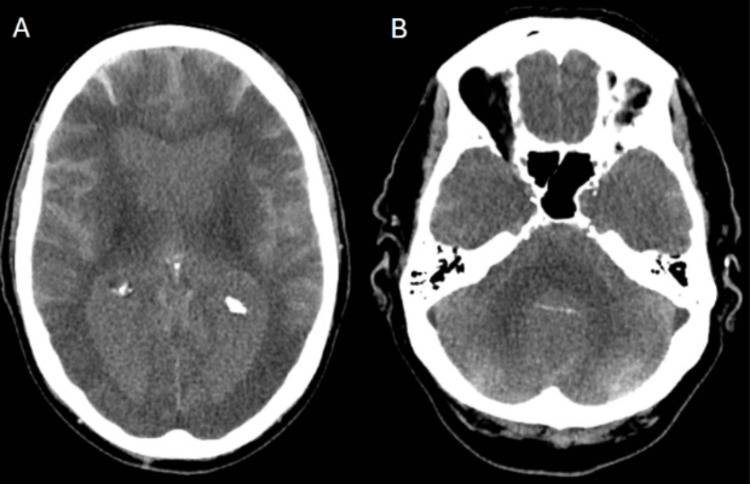
Single-energy CT Axial images (A, B) obtained from single-energy CT of the head, demonstrating hyperdensities in the ventricular and subarachnoid spaces.

**Figure 2:**
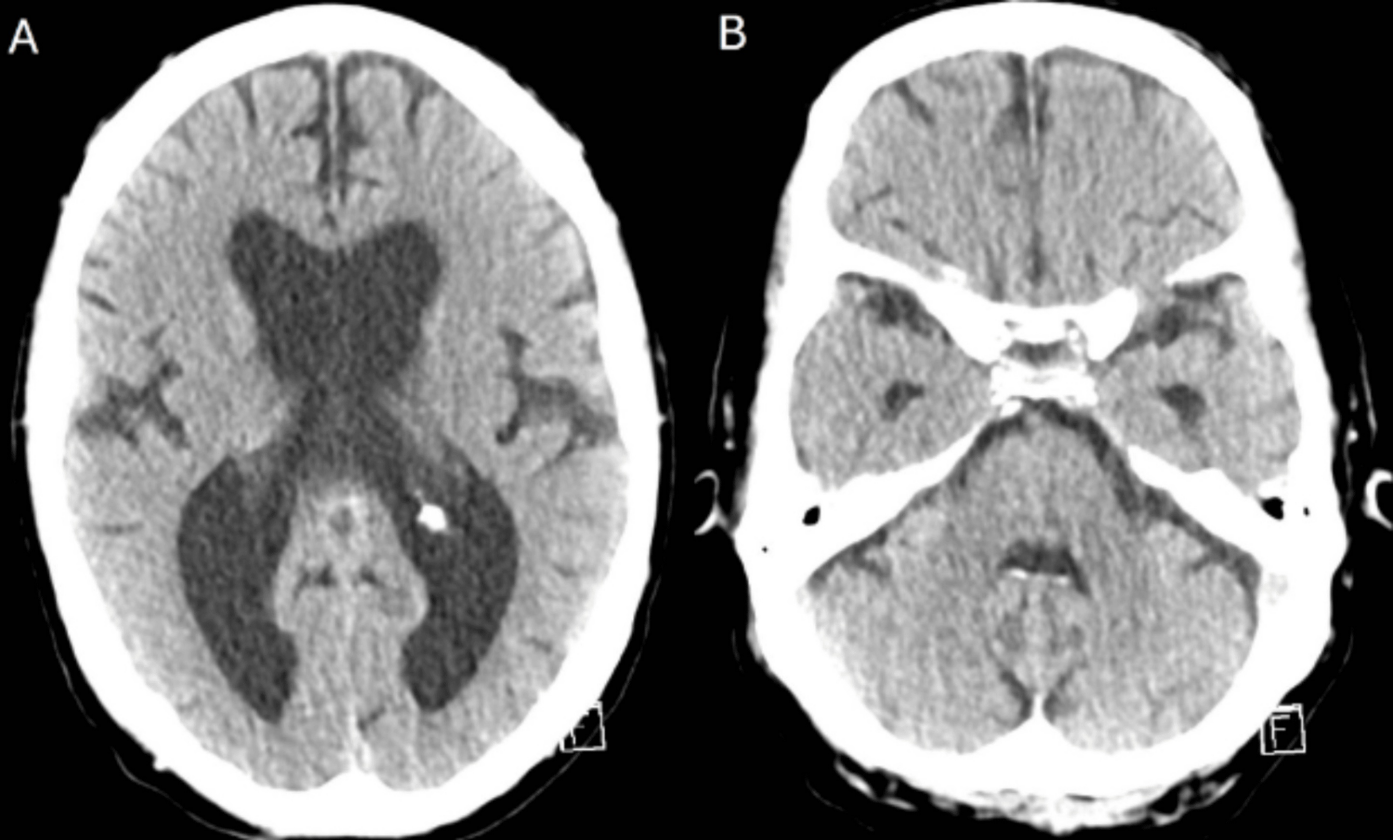
Dual-energy CT Axial images (A, B) were obtained from the dual-energy CT of the head, demonstrating the absence of hyperdensities within the ventricles and subarachnoid space.

**Figure 3:**
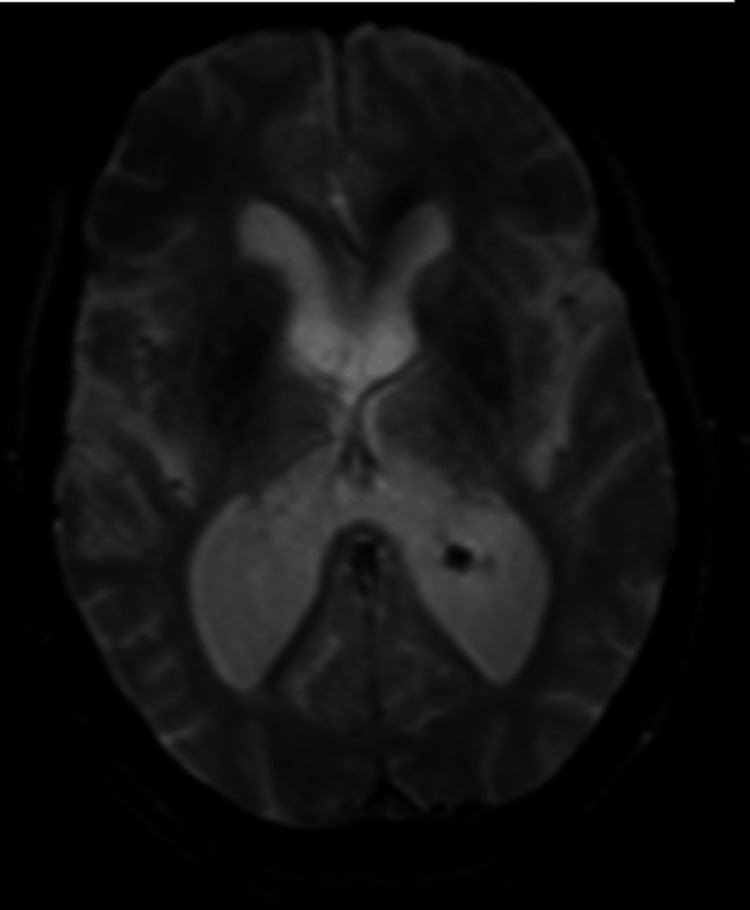
MRI An axial MRI image of the brain, obtained using a gradient echo (GRE) sequence, showing the absence of susceptibility artifacts within the ventricular and subarachnoid spaces.

Due to the scalp hematoma, a trauma evaluation was done including chest X-ray, pelvic X-ray, and a focused assessment with sonography for trauma (FAST) scan, all of which were negative. A repeat head CT showed the previous findings of potential IVH and SAH (Figure 1), and a CT angiography (CTA) of the head and neck showed no significant vascular abnormalities. The CT findings of IVH and SAH did not correlate with his physical exam, as the large amount of hemorrhage shown would likely cause the patient to be affected neurologically. An MRI brain without contrast and a DECT scan with iodine subtraction images were ordered. The results of the DECT with iodine subtraction (Figure 2) ruled out the IVH and SAH and confirmed that the hyperdensities were related to iodinated contrast despite the patient not remembering receiving any contrast dye. The absence of hemorrhage was also confirmed with the normal gradient echo (GRE) (Figure 3) sequences on MRI. The iodine contrast was possibly administered during his admission for compressive myelopathy at the private hospital for which physicians at our institution were unable to access medical records.

Furthermore, the diffuse cerebral edema shown on the initial CT was not present on follow-up DECT, MRI, or CTA. This finding was likely artifactual, related to the Mach effect from the contrast brain interface [9]. The patient remained in the hospital for several weeks before being discharged to a retirement home.

## Discussion

DECT uses two different energy spectra, either using two X-ray sources and two detectors or an advanced single X-ray source and detector [10]. Usually, energies used range from 80-100 kVp for the lower energy spectrum and 140-150 kVp for the higher energy spectrum [8]. Attenuation is defined as the reduction of the intensity of an X-ray beam as it traverses matter and photons become absorbed or deflected [11]. Attenuation is based on Compton scattering and the photoelectric effect. These are dependent on the energy of the X-ray beam, as well as the thickness and atomic number of the tissue, with higher atomic numbers correlating with a larger attenuation [8,12].

Attenuation coefficients in CT imaging are expressed in Hounsfield Units (HUs) [13]. Different materials can have similar HUs at a given energy. Blood and iodine have similar HUs in the energy range commonly used for SECT imaging [8]. As a result, it can be challenging for physicians to determine whether a hyperdensity on CT is hemorrhage or iodine contrast [14]. However, since the attenuation of materials decreases as energy increases [15], a material will have different HUs at different energy levels. In addition, the rate of change in HUs at different energy levels is not the same for different materials. Due to this variability, DECT allows for enhanced differentiation of materials.

Attenuation increases rapidly when the photon energy exceeds the binding energy of K-shell electrons; this effect is called the K-edge. DECT can use these sudden changes of attenuation at different energy levels to differentiate materials [8]. Furthermore, the DECT image-reconstruction algorithm can quantify the concentration of the elements within tissues: DECT can calculate the contribution of the iodine contrast within single voxels and reconstruct the data to create new images [8]. DECT can also subtract iodine, or calcium, from these blended images, resulting in a virtual non-contrast image (VNC). DECT’s ability to create a VNC led to the differentiation of iodine and hemorrhage in our case [8].

These capabilities of DECT allow it to differentiate iodine and hemorrhage within any compartment of the brain with high sensitivity and specificity [6]. Other benefits of DECT are its potential reduction of contrast agents and radiation doses, reduction of artifacts, and frequent improvement of contrast-to-noise and signal-to-noise ratios over conventional CT. Drawbacks include limited availability relative to SECT, increased purchase/maintenance cost, increased acquisition time, potential temporal misregistration, and potential decreased field of view [10].

Although DECT is effective in differentiating hemorrhage from iodine contrast, taking a detailed clinical history can be helpful for physicians with regard to this differentiation. In this case, the patient was unable to recall any recent iodine contrast administration, nor did he have any risk factors for blood-brain barrier disruption, leading multiple physicians to discount contrast staining in the differential and to suspect IVH and SAH instead. However, the patient’s lack of symptoms and nonfocal neurologic exam raised suspicion that IVH and SAH were not present. This diagnostic dilemma was definitively resolved with DECT.

MRI also continues to be an important tool in differentiating intracranial hemorrhage from iodine contrast agents, with the benefit of no radiation exposure [16]. However, the longer acquisition time of MRI can lead physicians to favor DECT in cases of suspected active hemorrhage, particularly if patient cooperation for the duration of MRI is difficult to achieve. Additionally, DECT would be useful for patients with implanted devices of unknown MRI compatibility. It is important to note that if a medical center does not perform either DECT or MRI procedures, CT scans must be performed at least 19-24 hours after suspected contrast administration to reliably differentiate iodine contrast from hemorrhage [17].

## Conclusions

This case report highlights the utility of DECT in differentiating IVH and SAH from iodine contrast agents in a complex clinical scenario. Hemorrhage and iodine have similar HU densities at the energy levels used in conventional SECT, so acquiring imaging at multiple energies allowed DECT to rule out hemorrhage in this case. Both DECT and MRI are able to differentiate intracranial hemorrhage versus iodine contrast agents. DECT has the advantage of more rapid image acquisition relative to MRI which is useful in emergency situations but has the disadvantage of radiation exposure. The choice of DECT and/or MRI modality to choose should be decided on a case-by-case basis.
